# Utilization of Immunotherapy for the Treatment of Hepatocellular Carcinoma in the Peri-Transplant Setting: Transplant Oncology View

**DOI:** 10.3390/cancers14071760

**Published:** 2022-03-30

**Authors:** Maen Abdelrahim, Abdullah Esmail, Ashish Saharia, Ala Abudayyeh, Noha Abdel-Wahab, Adi Diab, Naoka Murakami, Ahmed O. Kaseb, Jenny C. Chang, Ahmed Osama Gaber, Rafik Mark Ghobrial

**Affiliations:** 1 Section of GI Oncology, Department of Medical Oncology, Houston Methodist Cancer Center, Houston, TX 77030, USA; aesmail@houstonmethodist.org; 2 Cockrell Center of Advanced Therapeutics Phase I Program, Houston Methodist Research Institute, Houston, TX 77030, USA; 3 Department of Medicine, Weill Cornell Medical College, New York, NY 10065, USA; asahara@houstonmethodist.org (A.S.); jchang@houstonmethodist.org (J.C.C.); agaber@houstonmethodist.org (A.O.G.); rmghobrial@houstonmethodist.org (R.M.G.); 4 Houston Methodist Research Institute, Houston, TX 77030, USA; 5 JC Walter Jr Center for Transplantation, Sherrie and Alan Conover Center for Liver Disease and Transplantation, Hoston Methodist Hospital, Houston, TX 77030, USA; 6 Section of Nephrology, Division of Internal Medicine, The University of Texas MD Anderson Cancer Center, Houston, TX 77030, USA; aabudayyeh@mdanderson.org; 7 Section of Rheumatology and Clinical Immunology, Department of General Internal Medicine, Division of Internal Medicine, The University of Texas MD Anderson Cancer Center, Houston, TX 77030, USA; nhassan@mdanderson.org; 8 Department of Rheumatology and Rehabilitation, Faculty of Medicine, Assiut University Hospitals, Assiut University, Assiut 71515, Egypt; 9 Section of Melanoma Medical Oncology, Division of Cancer Medicine, The University of Texas MD Anderson Cancer Center, Houston, TX 77030, USA; adab@mdanderson.org; 10 Division of Renal Medicine, Brigham and Women’s Hospital, Harvard Medical School, Boston, MA 02115, USA; nmurakami@bwh.harvard.edu; 11 Section of GI Medical Oncology, Division of Cancer Medicine, The University of Texas MD Anderson Cancer Center, Houston, TX 77030, USA; akasb@mdanderson.org; 12 Section of Breast Oncology, Department of Medical Oncology, Houston Methodist Cancer Center, Houston, TX 77030, USA

**Keywords:** transplant oncology, liver transplantation, hepatocellular carcinoma, immunotherapy, immune checkpoint inhibitors, CTLA-4 inhibitors, PD-1 inhibitors, allograft rejection

## Abstract

**Simple Summary:**

Hepatocellular carcinoma is the second most common cause of cancer-related deaths and accounts for over eighty percent of primary liver cancers worldwide. Regarding the Milan Criteria, only a small portion of HCC patients are eligible for liver transplantation due to advanced-stage disease and large tumor size preventing/delaying organ allocation. Recently, the use of anti-programmed cell death protein 1 and programmed cell death ligand 1 (PD-1 and PD-L1) checkpoint inhibitors in the treatment of cancers have evolved rapidly and these therapies have been approved for the treatment of HCC, however, the main concerns about organ rejection in liver transplant patients who will be treated with ICPIs are still the same in both pre-and post-transplant setting. To alleviate those concerns, more global collaborations to explore the safety and efficacy of ICPIs in both the pre-and post-organ transplantation settings are required. The decision to administer ICPI treatment in liver transplant patients should be made on a case-by-case basis according to the goal of care and the availability and efficacy of other treatment options.

**Abstract:**

Hepatocellular carcinoma (HCC) represents the second most common cause of cancer-related deaths and accounts for over eighty percent of primary liver cancers worldwide. Surgical resection and radiofrequency ablation in small tumors are included in the treatment options for HCC patients with good liver function profiles. According to the Milan Criteria, only a small portion of HCC patients are eligible for liver transplantation due to advanced-stage disease and large tumor size preventing/delaying organ allocation. Recently, the use of anti-programmed cell death protein 1 and programmed cell death ligand 1 (PD-1 and PD-L1) checkpoint inhibitors in the treatment of cancers have evolved rapidly and these therapies have been approved for the treatment of HCC. Immune checkpoint inhibitors have resulted in good clinical outcomes in pre-and post-transplant HCC patients, although, some reports showed that certain recipients may face rejection and graft loss. In this review, we aim to illustrate and summarize the utilization of immune checkpoint inhibitor therapies in pre-and post-liver transplants for HCC patients and discuss the assessment of immune checkpoint inhibitor regulators that might determine liver transplant outcomes.

## 1. Introduction

Hepatocellular carcinoma (HCC) is the second most common cause of cancer-related deaths and accounts for over eighty percent of primary liver cancers worldwide [1,2,3,4]. Curative therapies options include surgical resection in patients with well-compensated liver function and radiofrequency ablation in small tumors; however, in ninety percent of patients HCC occurs in the setting of cirrhosis where optimal management remains liver transplantation with five-year survival rates of approximately eighty percent [4,5].

Liver transplantation has been an excellent option of treatment for HCC patients after Milan criteria were established in 1996 [4,6,7]. Milan Criteria was created by Mazzaferro et al. and defined as a model to determine the eligibility of patients with HCC for liver transplantation [4]. Milan criteria state that the tumor diameter of a single lesion is less than or equal to five centimeters or, for multiple lesions, no more than three tumors, each less than or equal to three centimeters, without vascular invasion or extrahepatic metastases. Despite the success of liver transplantation in treating HCC, only a small portion of patients fit into standard Milan Criteria to receive liver transplantation due to: (i) advanced-stage cancer and/or greater tumor size limiting organ placement, and (ii) a lack of neoadjuvant (bridging) medicines that successfully down-stage or delay tumor growth for patients waiting for a liver transplant. Several institutions have used AFP dynamics to assist decide who would benefit from downstaging techniques. Even though patients beyond the Milan criteria who have had a transplant have a comparable survival rate, there is a significantly greater incidence of drop-off from the waiting list, and survival in this cohort is extremely dismal. Patients are most typically downstaged to Milan criteria and transplanted using locoregional treatments (LRT). In 2007, the Food and Drug Administration FDA approved an antiangiogenic tyrosine kinase inhibitor (Sorafenib) as the first and only FDA-approved therapy for HCC in a decade. Starting in 2017, the FDA approved several other agents for advanced HCC, based on randomized phase III clinical trial data [8,9,10,11,12]. These treatments include regorafenib, cabozantinib, and ramucirumab in refractory disease and lenvatinib and atezolizumab/bevacizumab in the first-line treatment [8,9,10,11,12].

Atezolizumab and bevacizumab were shown to have response rates of 27 percent and 12 percent, respectively, in the IMbrave150 trial of atezolizumab and bevacizumab versus sorafenib. In the combination arm, the median survival was not achieved compared to thirteen months in the sorafenib alone arm (HR 0.58, 95 % CI 0.42–0.79; *p* = 0.0006) [10,13]. An antiangiogenic plus immune checkpoint inhibitor (ICPI) combination can reactivate intertumoral trafficking of cytotoxic T cells and generate a favorable immunological milieu for ICPI antitumoral action. This research has transformed the HCC therapy paradigm, recommending a new standard of care for intermediate-stage HCC patients who have failed to respond to local treatments and those with advanced-stage disease who are suitable for first-line treatment. This study emphasized the importance of systemic treatment in the management of HCC and challenges historical treatment approaches. It further emphasizes the need to maintain liver function so that patients can receive systemic treatments.

There is minimal evidence to support systemic therapy in the neoadjuvant setting as well as a bridging strategy to liver transplantation. Continued eligibility and transplant timing are critical, and they can be modified by three factors: donor availability, Model for End-Stage Liver Disease (MELD) score, and blood group. A recent case report indicated the feasibility of PD-1 blocking prior to orthotopic liver transplantation, with no signs of disease recurrence one year after transplantation. In this case report, nivolumab was discontinued six weeks before the transplant. Because atezolizumab has a half-life of 27 days and bevacizumab has a half-life of 20, it is necessary to discontinue taking both atezolizumab and bevacizumab at the same time point.

The chance of graft rejection is the main concern while using ICPI peri-transplant [14,15]. The immunological tolerance of the graft is thought to be facilitated by the programmed death 1 (PD-1) and cytotoxic T-lymphocyte-associated antigen-4 (CTLA4) pathways; PD-L1 is expressed in post-transplant liver allografts, and PD-1 is substantially expressed on graft-infiltrating T-cells [16,17]. CTLA4 binding to its counter-receptor B7 on T-cells produces an inhibitory signal that stops T-cell responses [17,18]. As a result, blocking these pathways may cause these T-cells to become more active, resulting in T-cell-mediated graft rejection. However, given the limited amount of research we’ve looked at, we believe there are some circumstances in which ICI use may result in a lower risk of graft rejection that we will discuss through this review.

## 2. ICPI Pre-Liver Transplant: HCC Bridging Therapy

Patients with HCC that have progressed beyond the Milan criteria are frequently treated locally with radiofrequency ablation, transarterial chemoembolization (TACE), or transarterial radioembolization [19]. Combining systemic and locoregional therapy to downstage these patients and make them eligible for LT is gaining popularity. However, there are limited studies on the role of neoadjuvant systemic treatment, such as ICPIs, in the era of pre-liver transplantation.

In the last two decades, liver transplantation became another treatment option for HCC patients, and it resulted in the impressive beneficial outcome of approximately eighty percent five-year survival rate [4]. However, a significant number of HCC patients remain ineligible for liver transplantation according to the standards of the Milan Criteria due to advanced-stage disease and/or large tumor size preventing/delaying organ allocation.

Immune checkpoint inhibitors (ICPIs), have achieved notable improvements against unresectable HCC, such as pembrolizumab, investigated as pembrolizumab versus placebo in a total of 278 and 135 patients who received pembrolizumab and placebo, respectively; this clinical trial showed the median OS was 13.9 months in the pembrolizumab arm compared to10.6 months in the placebo arm [20]. In the same direction, atezolizumab (ICPIs) plus bevacizumab (VEGF inhibitor) was reported a PFS of 6.8 months vs. 4.3 months in the sorafenib arm as well as the median OS was 19.2 months vs. 13.4 months, respectively [10,21]. In addition, nivolumab plus ipilimumab (both ICPIs), this combination was investigated with a total of 148 patients and showed a promising median OS of 22.8 months [22].

Recently, interventions to downstage the disease or delay tumor progression for patients on the liver transplantation waiting list have been achieved effectively by neoadjuvant agents. Some studies revealed that in some patients ICPI therapy resulted in numerous adverse events. Other studies showed that these therapies can be well tolerated, with only fifteen percent of patients with unresectable HCC experiencing adverse events that required treatment discontinuation. It was reported that nine patients were listed and successfully transplanted after treatment with ICPIs, such as nivolumab, in the bridging setting from 2017 to 2020 [23]. The mean age of the nine patients was fifty-seven years and sixty-seven percent of them were male. Five patients (56%) had records of HCC resection at a median interval of seven years and one transplant (11%) was from a living donor. The nine patients received ICPIs (Nivolumab) every two weeks and eight (89%) patients received their last dose within 4 weeks of transplantation. In addition, immunosuppression was administered as steroids with an initial dose of five-hundred milligrams methylprednisolone tapered to prednisone (10 mg/day) over two weeks together with mycophenolate mofetil and tacrolimus. Post-transplant, the patients were followed up at a median of sixteen months with a range from eight to twenty-three months. Pathological outcomes revealed near-complete (more than 90%) tumor necrosis in one-third of the cases. Additionally, no severe allograft rejections/losses, tumor recurrences, or deaths occurred except in one patient who had mild acute rejection as a result of a low dosage of tacrolimus (less than 6 ng/mL) and responded rapidly to increased dosage.

In 2019, a study by Feun et al. showed that twenty-eight out of twenty-nine patients were evaluable for response in a Phase 2 Study of Pembrolizumab in unresectable HCC [5]. One patient achieved a complete response and eight patients achieved partial responses for a total response rate of thirty-two percent. Four other patients reported stable disease. The median progression-free survival was four and half months and the median overall survival was thirteen months. PD-L1 and plasma PD-L1/PD-1 levels were linked with plasma IFN-γ or IL-10, where the most common adverse events were increases in aspartate aminotransferase with or without increases in alanine aminotransferase and serum bilirubin. Generally, the toxicities were tolerable and reversible. The preliminary findings of the study indicated that baseline TGF-β plasma levels may be a predictor of response. [24] Since some studies suggest that the use of ICPIs in the liver transplantation setting may potentially lead to high rates of graft loss due to possible dysregulation of immune activation, the use of ICPIs remains controversial. In the future, more in-depth collaborations between transplant oncologists, transplant teams (surgeons and transplant hepatologists, and immunologists may prove beneficial for the design of optimal treatment regimens.

The use of ICPIs as bridging therapy is a steadily evolving field that requires more clinical trials to clearly understand the optimal approach for ICPIs’ use in patients waiting for liver transplantation and to better predict risk while minimizing graft loss rate. Ongoing clinical trials at our institution and others are on the way to further clarify the feasibility of using ICPI prior to liver transplantation.

## 3. ICPI Post-Liver Transplant: HCC Palliative Therapy

Although immune therapy after a solid organ transplant was previously assumed to be inappropriate due to the risk of allograft rejection, new studies have demonstrated that LT recipients can be treated with ICPIs in the appropriate circumstances Table 1. Nearly two-thirds of LT recipients treated with ICPIs had their allografts preserved, according to recent data [25]. These studies looked at 48 organ transplant patients who had advanced cancer and were given ICPI. In this report, 19 of these patients had liver transplants, 10 of these 19 patients were diagnosed with HCC. The disease control rate was reported to be 21%, while 37% of liver transplant patients experienced graft rejection.

Munker and DeToni conducted another study in which they reviewed reports on 14 known cases of liver transplant recipients who received ICPI treatment [26]. One factor that could influence susceptibility to organ rejection was the choice of an immunosuppressive agent. The presence of PDL-1 in liver graft biopsies, as well as the time of treatment initiation only four of the fourteen reported cases of liver graft rejection were reported in this report (28 percent). The median time to rejection was three weeks after the start of immune therapy. Regarding this study, OS was reported in 12 patients, with a median duration of 1.2 months. Surviving time in four patients who responded to treatment ranged from four to eighteen months [26].

Furthermore, Rammohan et al. [27] illustrated a case of HCC that was originally treated with a liver transplant but progressed to lung 3 years later. After failing to respond to sorafenib, the patient was given an ICPI (pembrolizumab). In combination with sorafenib. The patient remained healthy ten months after commencing ICPI and was treated with the combination of pembrolizumab and sorafenib with no reports of tumor or graft rejection/dysfunction [27]. Similarly, De Bruyn et al. published a study on 19 liver transplant cases who were received ICPIs to treat advanced malignancies. This study found that 21% of patients had disease control and 38% had graft rejection, implying that ICPIs can be used to treat liver transplant recipients [25]. In addition, Abdel-Wahab et al. reported 39 cases with allograft transplantation, finding that the median period of ICPI initiation (combined anti-CTLA-4 and anti-PD-1 treatment) was nine years after transplantation in the 28 percent of patients (11 out of 39) with LT. Allograft rejection was reported in just 41% of all patients enrolled, and in the hepatic patients, only four out of 11 developed allograft rejection. These papers do not provide enough evidence to conclude that one ICPI or immunosuppressant agent is safer than another. However, it was proposed that liver biopsies of liver allografts be performed routinely before treatment commencement in LT recipients, and that, in the absence of contraindications, pre-treatment with steroids be undertaken, and immunosuppression be gradually tapered under strict supervision.

Currently, the utility of ICPI post-LT is being investigated as a treatment approach for HCC patients with disease recurrence or in the setting of second primary cancer that is eligible for ICPI therapy. However, as of the date of this review, there is not enough data on safety or to support the use of ICPI after liver transplantation. In addition, the link between graft rejection and tumor response is still unknown. There are few predictive biomarkers for adapting immunotherapy for HCC patients after LT [28,29]. Immunotherapy, on the other hand, has gained increased acceptance in the transplant oncology community as a bridge therapy to LT [30]. More prospective data will be required in the future to support its safety and efficacy.

**Table 1:** Summary of thirteen case reports of the use of immune checkpoint inhibitors (ICPIs) in the post-liver transplant setting as palliative therapy for patients with HCC; **ICPI:** Immune Checkpoint Inhibitors, **M:** male, **F:** female, **PD-1:** Programmed Death, **mg:** milligram, **D:** death, **MMF:** Mycophenolate mofetil, **UK:** unknown, **IST:** immunosuppressive therapy, **PD:** a progressive disease. **OF**: organ failure.

Age/Sex	ICPIs Agent	ICPIs Cycles	ICPIs Class	Interval Time from Transplant to ICPIs	IST	Type of Response	Graft Outcome	References
70 M	Nivolumab	(4)	PD-1	33 Months	Tacrolimus/high-dose steroids.	PD	No rejection	Al Jarroudi et al. [31]
62 F	Nivolumab	(5)	PD-1	12 Months	Tacrolimus	PD	No rejection	Al Jarroudi et al. [31]
66 M	Nivolumab	(6)	PD-1	24 Months	Tacrolimus	PD	No rejection	Al Jarroudi et al. [31]
56 M	Nivolumab	(6)	PD-1	31 Months	Tacrolimus	PD	No rejection	DeLeon et al. [32]
55 M	Nivolumab	(5)	PD-1	92 Months	Sirolimus/MMF	PD	No rejection	DeLeon et al. [32]
34 F	Nivolumab	UK	PD-1	43 Months	Tacrolimus	PD	No rejection	DeLeon et al. [32]
63 M	Nivolumab	UK	PD-1	14 Months	Tacrolimus	UK	No rejection	DeLeon et al. [32]
68 M	Nivolumab	UK	PD-1	13 Months	Sirolimus	UK	Graft rejection	DeLeon et al. [32]
41 M	Nivolumab	(15)	PD-1	16 Months	Tacrolimus (1 mg)	PD	No rejection	De Toni and Gerbes et al. [33]
70 M	Pembrolizumab		PD-1	96 Months	Low-dose (50%) Tacrolimus	PD	No rejection	Varkaris et al. [34]
53 F	Nivolumab	(1)	PD-1	36 Months	Everolimus/MMF	D due to OF (2 weeks after start ICPI)	Graft rejection	Gassmann et al. [35]
14 M	Nivolumab	(1)	PD-1	36 Months	Tacrolimus (4 mg)	D due to OF (5 weeks after start ICPI)	Graft rejection	Friend et al. [36]
20 M	Nivolumab	(2)	PD-1	48 Months	Sirolimus (2 mg)	D due to OF (4 weeks after start ICPI)	Graft rejection	Friend et al. [36]

## 4. Assessment of Immune Checkpoint Inhibitors

### 4.1. Does Immunosuppression Affect the Efficacy of ICPIs?

**Figure 1:**
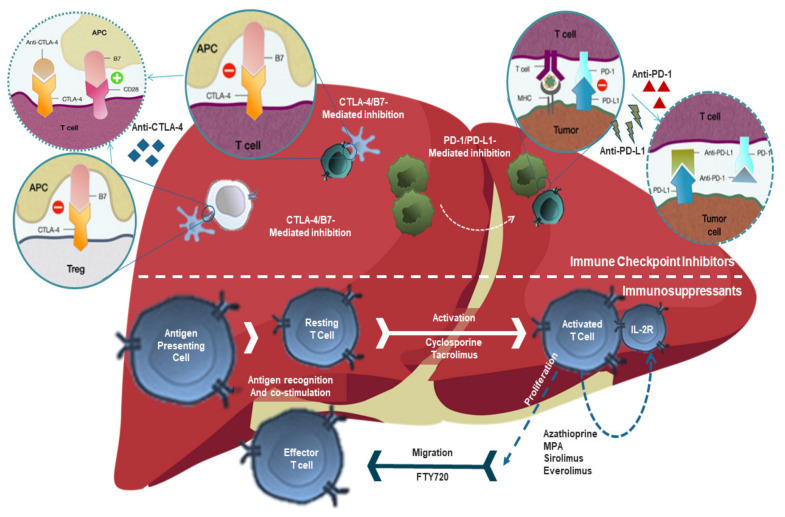
The mechanism of action of immunosuppressants versus immune checkpoint inhibitors. The mechanism of action of checkpoint inhibitors is to prevent the “off” signal from being sent, allowing the T cells to kill cancer cells. Such drugs that act against the checkpoint proteins are called CTLA-4 inhibitors, PD-1 inhibitors, and PD-L1 inhibitors. While the mechanism of action of immunosuppressants is that they all function to prevent allograft rejection by preventing/inhibiting cell activation, cytokine production, differentiation, and/or proliferation. **ICPI:** Immune Checkpoint Inhibitors, **PD-1:** Programmed Death-1, **IST:** immunosuppressive therapy, **APA**, Antigen-presenting cell; **CTLA-4**, cytotoxic T-lymphocyte–associated antigen 4; **PD-L1**, Programmed death-ligand 1.

Since the mechanism of action of immunosuppressant treatment highlighted in Figure 1, in theory, is against the intended effects of ICPIs in non-transplanted patients, it is an area that needs to be explored and investigated for liver transplants candidates and recipients who is receiving ICPI treatment. Immunosuppressants may have unwanted effects on these patients by reducing the efficacy of ICPIs that require a normal T-cell response for proper function Figure 1.

Recent data emphasized that concomitant use of steroids does not antagonize the result of ICPIs treatment [37]. Furthermore, other case reports showed that immunosuppression agents, such as Tacrolimus did not avert the response to ICPIs in four patients [32,33,38], Tacrolimus works by attaching to the immunophilin FKBP-12 (FK506 binding protein) and forming a novel complex that inhibits peptidyl-prolyl isomerase activity that results in inhibiting both T-lymphocyte signaling and IL-2 transcription. Moreover, another report revealed that patients who received high-dose of immunosuppressants (steroids) as a preventive treatment in order to avert the rejection from ICPIs have shown the same remarkable response in a solid organ transplant setting [39]. In another hand, several case reports have shown that using a sub-therapeutic dosage of immunosuppressants, such as Tacrolimus was not related to organ rejection [33,38,40].

These results suggest that using immunosuppression is a viable option, but screening will be required if there are any contraindications. Dose adjustments during the course of treatment can be conducted whenever it is needed.

### 4.2. Which ICPI Class Is Safer?

Different ICPIs act through different pathways and have specific mechanisms of action. For example, the ICPI ipilimumab is a cytotoxic T-lymphocyte-associated Protein 4 (CTLA4) inhibitor, whereas nivolumab and pembrolizumab are programmed cell death 1 (PD-1) and Programmed cell death 1 ligand 1(PD-L1) blocking agents. The mechanism of action of all ICPI classes has been illustrated in Figure 1.

At this time, there is not enough data available to support the use of a particular ICPI agent in favor of another when it comes to assessing the risk of causing liver rejection. In a study of liver transplants patients who have been treated with ICPIs, Stefan Munker et al. reported that of fourteen patients, only two were treated with a CTLA-4 inhibitor, such as ipilimumab, and the rest of the patients received either PD1 or PD-L1 blocking agents, such as nivolumab and pembrolizumab [26].

In other case reports, where immunosuppression was diminished to subtherapeutic levels, four out of ten patients who had organ rejection were treated with PD-1 medications, such as nivolumab or pembrolizumab, whereas no rejection was evident in two patients who were treated with a CTLA-4 inhibitor, such as ipilimumab [32,33,34,36,40,41,42,43]. Some authors attributed the worse outcomes in organ rejection in the patients who received PD1/PD-L1 treatment, to the predominant role of PD-1 in determining graft tolerance. In contrast, a case report showed that kidney graft rejections occurred in those who were treated with either CTLA-4 or PD-1 inhibitors.

Further studies are needed before we can confidently conclude that CTLA 4 inhibitors cause fewer rejections than PD-1/PDL-1 inhibitors in transplant recipients. As a result, there is currently no evidence to support the use of specific ICPIs in liver allograft recipients, and it appears reasonable that choosing ICPI agents should be guided by data available on effectiveness in the respective tumor types.

### 4.3. Can Liver Biopsy Help with Patient Selection and Prediction of Rejection?

Available reports show that liver biopsies might serve for the prognosis of rejection, as well as in helping the assessment to predict the ICPIs’ treatment response in some liver transplants patients. Data obtained from biopsies of three patients who had organ rejection showed positive PD-L1 staining, whereas PD-L1 staining was negative from four patients who had successful organ transplants without rejection. These results indicate that biopsy results with positive PD-L1 staining might predict organ rejection. It is advocated that liver biopsy, conducted as a component of patient assessment for those who undergo liver transplantation and will be treated with ICPIs, may help in choosing the proper ICPI, whether a PD1/PDL-1- or a CTLA4 blocking agent. It was proposed that liver biopsies of liver allografts should be conducted before treatment choice and if the liver biopsy reveals expression of high levels of PD-L1, a CTL4- inhibitor can be considered in the treatment plan. However, we believe this is a premature conclusion and more clinical data is needed.

### 4.4. The Timing of ICPI Peri-Transplant

**Figure 2:**
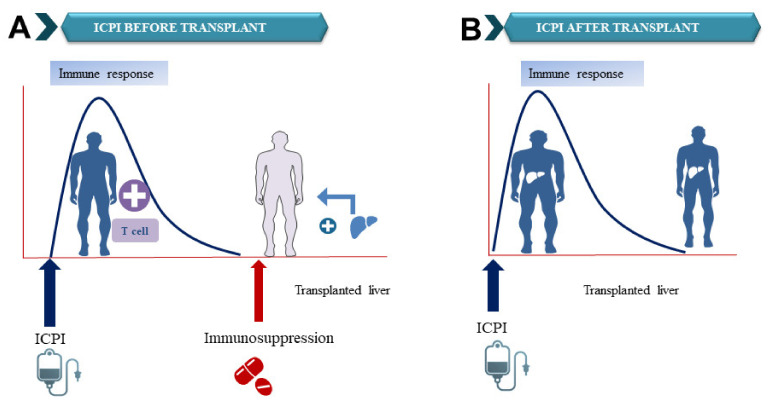
The expected timeline for using Immune checkpoint inhibitors in liver transplantation patients with HCC. (**A**) ICPI will free the immune system and active T-cell to attack and kill cancer cells. This state of the “hyperactive” immune system occurs in the absence of the allograft. Once ICPI is held, the immune system will “cool off” and return gradually to the normal body immune state at which a new liver transplant can be achieved safely.(**B**) ICPI can be used post-transplant to treat cancer recurrence or new second primary cancer. In this setting, the immune system will be activated in the presence of the graft, so the risk of graft rejection will be higher at around 30% than the general population.

There are two scenarios where ICPI can be used in liver transplant candidates or recipients Figure 2. In the first scenario, ICPI will be used in the pre-transplant setting in liver transplant recipients as bridging or neoadjuvant therapy Figure 2A. In this setting, ICPI will unleash the immune system and active T-cell to attack and kill cancer cells. This state of the “hyperactive” immune system occurs in the absence of the allograft. Once ICPI is held, the immune system will “cool off” and return gradually to the normal body immune state at which a new liver transplant can be achieved safely. In addition, the use of significant immunosuppressants peri-transplant will further dampen the immune system to protect the graft from the recipient’s own immune system which will further neutralize the effect of ICPI during this crucial time for the new allograft. An appropriate time interval between the last dose of immunotherapy and the liver transplant will further support the safety of ICPI before transplant. The ideal time is yet to be determined; however, 4–12 weeks have been reported.

Plasma concentrations of any given drug usually decline under clinically significant amounts after three half-lives. We anticipate that the best time for a liver transplant following the last dosage of ICPI would be at least three half-lives of the ICPI in use, which would put the patient outside of the risk period for the majority of liver-related complications, including liver allograft rejection or toxicity. Nonetheless, any long-term impact of ICPIs following plasma removal must be identified. A clinical trial is currently conducted at our institution and others to determine the feasibility of employing ICPI prior to liver transplantation.

In the second scenario Figure 2B, immunotherapy can be used post-transplant to treat cancer recurrence or new second primary cancer. In this setting, the immune system will be activated in the presence of the graft. The risk of graft rejection will be higher than the general population which was recently reported at around 30% [19]. A Clinical trial is currently conducted at our institution (Houston Methodist Cancer Center and JC Walter Jr Center for Transplantation) to determine the feasibility of employing ICPIs before liver transplantation (NCT05185505) [41].

### 4.5. What about Adverse Events of ICPIs?

ICPIs agents have been linked to a variety of side effects known as immune-related adverse events (irAEs) [44]. These side effects are typically thought to be separate from those associated with standard chemotherapy. The infiltration of activated T cells into normal tissues causes the irAEs produced by ICPI treatment. Although ICPI treatment has the potential to influence a variety of organ systems, nevertheless previous ICPI trials in HCC have indicated that these medicines are generally well tolerated, in addition, in Phase I/II and Phase III trials, Nivolumab showed an acceptable safety profile as well [45,46]. Pembrolizumab and durvalumab, the only anti-PD-L1 monoclonal antibody being tested in HCC, have similar safety findings [47], however, only Phase I/II trial data is available [48]. It has also been hypothesized that combining medicines with diverse molecular targets can improve antitumor response. Treatment-related adverse events with grade 3/4 were reported in 38% of HCC patients who received a combination of nivolumab and ipilimumab therapy. However, only 5% of patients, required treatment discontinuation [49]. In the same direction, in 56 percent of patients treated with the recently FDA-approved atezolizumab plus bevacizumab, grade 3/4 adverse events occurred, but only a few needed treatment terminations [50].

One of the most concerning irAEs associated with ICPIs usage is hepatotoxicity. Hepatitis is the most frequent type of liver injury, characterized by a significant increase in aminotransferases together with or without an increase in bilirubin levels. The development of liver injury usually occurs within a few weeks of starting medication. The degree of aminotransferase and bilirubin increase is related to the severity of hepatitis. In several clinical trials, the prevalence of hepatic damage associated with the use of ICPI as monotherapy ranged from 2% to 10% [51]. The occurrence of severe liver toxicity (Grade 3/4) is uncommon. On the other hand, combination ICPIs therapy has been linked to a higher rate of hepatotoxicity [52], which is likely due to anti-CTLA4 therapy, which is believed to be less tolerated than PD-1 or PD-L1 inhibitors [53]. Regarding these concerns of hepatotoxicity, high-dose corticosteroids are commonly used to treat severe hepatotoxicity, and the majority of patients respond well to this treatment [54,55].

## 5. Conclusions

ICPI therapies have tolerable side effects and excellent responses in the treatment of cancer patients as well as pre-transplant patients in the bridging therapy setting. In contrast, for post-transplant patients in the palliative setting, the existing data have eliminated the contraindication of using ICPIs in liver transplant patients. However, the main concerns about organ rejection in liver transplant patients who will be treated with ICPIs are still the same in both pre-and post-transplant settings. To alleviate those concerns, more global collaborations to explore the safety and efficacy of ICPIs in the pre-and post-organ transplantation settings are required. Finally, the decision to administer ICPI treatment in liver transplant patients should be made on a case-by-case basis according to the goal of care and the availability and efficacy of other treatment options. Biopsies of liver allografts might be used to predict rejection and decide the proper ICPI class to be used based on PD-1/PDL-1 expression; however, larger and prospective studies are missing to support this conclusion. The role and type of immunosuppression in the setting of peri-transplant use of ICPI are not defined yet whether one kind can be safer than others is yet to be decided. ICPI treatment is an evolving and promising therapeutic option in oncology. Further investigations of these agents in the pre-and post-transplant settings are highly needed.
